# Time Course of Optical Quality and Intraocular Scattering after Refractive Lenticule Extraction

**DOI:** 10.1371/journal.pone.0076738

**Published:** 2013-10-16

**Authors:** Kazutaka Kamiya, Kimiya Shimizu, Akihito Igarashi, Hidenaga Kobashi

**Affiliations:** Department of Ophthalmology, University of Kitasato School of Medicine, Kanagawa, Japan; University of Missouri-Columbia, United States of America

## Abstract

**Purpose:**

To assess the time course of optical quality and intraocular scattering in relation to visual acuity after femtosecond lenticule extraction (FLEx) for the correction of myopia.

**Methods:**

This study evaluated 36 eyes of 36 patients with spherical equivalents of −4.38±1.53 D [mean ± standard deviation] who underwent FLEx. Before surgery, and 1 week and 1, 3 and 6 months after surgery, we assessed the modulation transfer function (MTF) cutoff frequency, Strehl ratio, objective scattering index (OSI), and OQAS values (OVs), using a double-pass instrument. We also investigated the relationship of the OSI with corrected distance visual acuity (CDVA) preoperatively and postoperatively.

**Results:**

The mean changes in MTF cutoff frequency, Strehl ratio, OSI, OV100%, OV20%, and OV9% preoperatively and 6 months postoperatively were −5.51±15.01, −0.03±0.07, 0.35±0.83, −0.17±0.48, −0.14±0.38, and −0.09±0.22, respectively. We found no significant preoperative correlation between the OSI and logMAR CDVA (Spearman rank correlation coefficient r = 0.068, p = 0.69), and modest, but significant correlations 1 week and 1, 3, and 6 months postoperatively (r = 0.572, r = 0.562, r = 0.542, r = 0.540, p<0.001, respectively).

**Conclusions:**

FLEx induced a transient decrease in optical quality in association with an increase in intraocular scattering in the early postoperative period, possibly due to mild interface haze formation, but gradually recovered with time. It is suggested that this transient degradation in optical quality related to an increase in the intraocular scattering may result in a slight delay of CDVA recovery in the early postoperative period.

## Introduction

The femtosecond laser is one of the most revolutionary technologies in medical care in recent years. [1]–[3] This femtosecond laser allows very precise ablation with less thermal damage to the tissues than occurs with other lasers. It has primarily been utilized as an alternative to the microkeratome for making corneal flaps in laser in situ keratomileusis (LASIK). A recent breakthrough in this laser technology has resulted in a novel refractive procedure called refractive lenticule extraction (ReLEx), which requires neither a microkeratome nor an excimer laser, but uses only the femtosecond laser system as an all-in-one device for flap and lenticule processing. [4], [5] First clinical results with laser-induced extraction of a refractive lenticule were reported in highly myopic eyes, [4] and in blind or amblyopic eyes. [5] Furthermore, the ReLEx technique, which can be used for femtosecond lenticule extraction (FLEx) by lifting the flap and by small incision lenticule extraction (SMILE) without lifting the flap, has been proposed as an alternative to conventional LASIK for the correction of refractive errors. [6]–[17] Interestingly, visual acuity recovery following this novel technique has been reported to be slightly slower than that after other keratorefractive surgeries in the early postoperative period, which may be a unique characteristic of this surgical approach. [11], [12], [15]–[17] This delay was considered to be mostly associated with interface haze formation, which is one of the most common adverse events after this surgery. However, to the best of our knowledge, there have so far been no clinical studies on quantitative assessment of the detailed optical quality including the intraocular scattering after this novel surgical procedure. The purpose of this study is twofold: to retrospectively assess the time course of the detailed optical quality of the eye, including the intraocular scattering, and to investigate the relationships of the intraocular scattering with visual acuity, after FLEx for the correction of myopic refractive errors.

## Patients and Methods

### Study Population

Thirty-six eyes (10 of men and 26 of women) of 36 consecutive patients who underwent FLEx for the correction of myopia and myopic astigmatism using the VisuMax femtosecond laser system (Carl Zeiss Meditec, Jena, Germany) with a 500 kHz repetition rate, were included in this retrospective study. Only one eye was selected randomly for statistical analysis in subjects undergoing bilateral FLEx. The sample size in this study offered 83% statistical power at the 5% level in order to detect a 0.50-difference in the objective scattering index (OSI) between two groups, when the standard deviation of the mean difference was 1.00, and offered 92% statistical power at the 5% level in order to detect a correlation of 0.50. The inclusion criteria for this surgical technique in our institution were as follows: unsatisfactory correction with spectacles or contact lenses, manifest spherical equivalent −9 diopters (D) or less, manifest cylinder 4 D or less, sufficient corneal thickness (estimated total postoperative corneal thickness >400 µm and estimated residual thickness of the stromal bed >250 µm), endothelial cell density ≥1800 cells/mm^2^, no history of ocular surgery, severe dry eye, progressive corneal degeneration, cataract, or uveitis. Eyes with keratoconus were excluded from the study by using the keratoconus screening test of Placido disk videokeratography (TMS-2, Tomey, Nagoya, Japan). The patient age at the time of surgery was 31.0±5.5 years (mean age ± standard deviation; range, 20 to 41 years). The preoperative manifest spherical equivalent was −4.38±1.53 D (range, −1.50 to −7.50 D). The preoperative manifest refractive cylinder was −0.62±0.66 D (range, 0.00 to −2.75 D). In all eyes, the preoperative manifest refraction was selected as the target correction. Routine postoperative examinations, including the usual slit-lamp biomicroscopic and funduscopic examinations, were performed 1 day, 1 week, and 1, 3, and 6 months after surgery. The study was approved by the Institutional Review Board of Kitasato University and followed the tenets of the Declaration of Helsinki. Written informed consent was obtained from all patients after explanation of the nature and possible consequences of the study.

### Surgical Procedure

FLEx was performed using the VisuMax femtosecond laser system (Carl Zeiss Meditec AG) with a 500 kHz repetition rate. The laser was visually centered on the pupil. A small (S) curved interface cone was used in all cases. In order, the main refractive and nonrefractive femtosecond incisions were performed in the following automated sequence: the posterior surface of the lenticule (spiral in pattern), the anterior surface of the lenticule (spiral out pattern), followed by a side cut of flap. The femtosecond laser parameters were as follows: 120 µm flap thickness, 7.5 mm flap diameter, 6.5 mm lenticule diameter, 140 nJ power for lenticule and flap, spot distance 3 µm, track distance 3 µm, with side cut angles at 90°. After completion of the laser sequence, a Siebel spatula was inserted under the flap near the hinge and the flap was lifted, the refractive lenticule was then grasped with forceps and extracted. The flap was then repositioned. All surgeries were uneventful and no definite intraoperative complication was observed. After surgery, steroidal (0.1% betamethasone, Rinderon^ TM^, Shionogi, Osaka, Japan) and antibiotic (0.5% levofloxacin, Cravit^TM^, Santen, Osaka, Japan) medications were topically administered 4 times daily for 2 weeks, and then the frequency was steadily reduced.

### Optical Quality Measurement

Before surgery, and 1 week and 1, 3, and 6 months after surgery, we measured the optical quality parameters of the eye, such as the modulation transfer function (MTF) cutoff frequency, the Strehl ratio, the OSI, and the OQAS values (OVs) (100%, 20%, and 9%) using the Optical Quality Analysis System (OQAS, Visiometrics, Terrassa, Spain) for a 4.0-mm pupil. [18]–[22] The manifest refractive error of the subjects was fully corrected during these measurements; the spherical error (up to −8.00 D) was automatically corrected by the double-pass system, and the residual spherical error (over −8.00 D) as well as the cylindrical error was corrected with an external lens, because the uncorrected refractive error directly affects the optical outcome of the system. The pupil diameter was provided by this device from an image of an additional video camera that allowed pupil alignment. Since these optical quality parameters are known to be affected by pupil diameter, [23]–[25] we confirmed that the pupil diameter was more than 4.0 mm in all eyes. The room illumination was kept low (approximately 25 lux) during testing. The value considered is the cutoff point of the MTF curve on the x-axis; the results are given in cycles per degree, representing the highest spatial frequency at lower contrast. The MTF cutoff in the double-pass system is the frequency at which the MTF reaches a value of 0.01. Because the point spread function (PSF) images recorded by the double-pass instrument can be affected by high-frequency noise, which is inherent in the use of cameras, the frequency for very small MTF values may become unstable, potentially leading to artifacts. To avoid this problem, the device uses an MTF threshold value of 0.01, which corresponds to 1% contrast. Thus, the MTF cutoff frequency in this article refers to the frequency up to which the eye can focus an object on the retina with a significant 1% contrast. The Strehl ratio is an expression of the ratio of the central maximum of the illuminance of the PSF in the aberrated eye to the central maximum that would be found in a corresponding aberration-free system. It is the measure of the fractional drop in the peak of the PSF as a function of the wavefront error. The OSI is an objective evaluation of intraocular scattered light. The index is calculated by evaluating the amount of light outside the double-pass retinal intensity PSF image in relation to the amount of light on the center. In the particular case of the instrument OQAS, the central area selected was a circle of a radius of 1 minute of arc, while the peripheral zone was a ring set between 12 and 20 minutes of arc. [21], [22] The OSI for normal eyes would range around 1, while values over 5 would represent highly scattered systems. The three OVs are normalized values of three spatial frequencies, which correspond to MTF values that describe the optical quality of the eye for three contrast conditions, commonly used in ophthalmic practice: 100% (OV 100%), 20% (OV 20%), and 9% (OV 9%). Specifically, the OV100% is directly related to the MTF cutoff frequency (it is the MTF cutoff frequency divided by 30 cycles/degree) and, therefore, to the patient’s visual acuity, although it is not affected by retinal and neural factors. The OV 20% and OV 9% are computed in the same way from smaller frequencies that are linked to 0.05 and 0.1 MTF values, respectively, which maintain the proportion of contrasts of 20% and 9%. We also investigated the relationships of the OSI with logarithm of the minimal angle of resolution (logMAR) of corrected distance visual acuity (CDVA) before surgery, and 1 week and 1, 3, and 6 months after surgery.

### Statistical Analysis

All statistical analyses were performed using StatView version 5.0 (SAS, Cary, USA). One-way analysis of variance (ANOVA) was used for the analysis of the time course of changes, with the Dunnett test for multiple comparisons. A Spearman rank correlation test was used for statistical analysis to assess the relationships between the OSI and visual acuity. Unless otherwise indicated, the results are expressed as mean ± standard deviation, and a value of p<0.05 was considered statistically significant.

## Results

### Patient Population

Preoperative patient demographics are summarized in Table 1. No eyes were lost during the 6-month follow-up in this series. All surgical procedures were uneventful, and mild interface haze and optically insignificant microstriae developed in 4 eyes (11%) and 2 eyes (6%), respectively, during the first postoperative week. All these eyes were followed without surgical intervention, and gradually resolved thereafter. No epithelial ingrowth, diffuse lamellar keratitis, keratectasia, or any other vision-threatening complications were seen at any time during the 6-month observation period.

**Table 1:** Preoperative demographics of the study population.

Demographic Data	
Age (years)	31.0±5.5 years (range, 20 to 41 years)
Gender (% female)	72%
LogMAR UCVA	1.13±0.24 (range, 0.30 to 1.40)
LogMAR CDVA	−0.23±0.06 (range, −0.30 to −0.18)
Manifest spherical equivalent (D)	−4.38±1.53 D (range, −1.50 to −7.50 D)
Manifest cylinder (D)	−0.62±0.66 D (range, 0.00 to −2.75 D)
Mean keratometric reading (D)	43.4±1.1 D (range, 41.5 to 46.0 D)
Pupil diameter (mm)	5.8±0.8 mm (range, 4.1 to 7.2 mm)

LogMAR = logarithm of the minimal angle of resolution, UDVA = uncorrected distance visual acuity, CDVA = corrected distance visual acuity, D = diopters.

**Figure 1:**
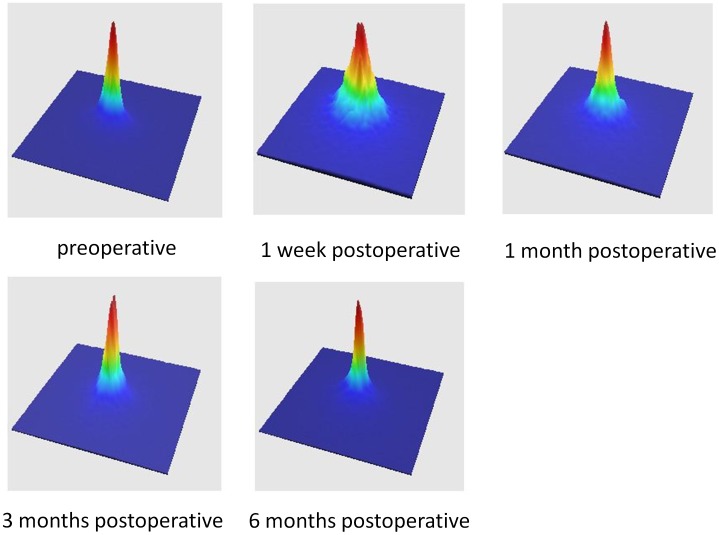
The representative point spread function images of eyes undergoing femtosecond lenticule extraction preoperatively, and 1 week and 1, 3 and 6 months postoperatively.

**Figure 2:**
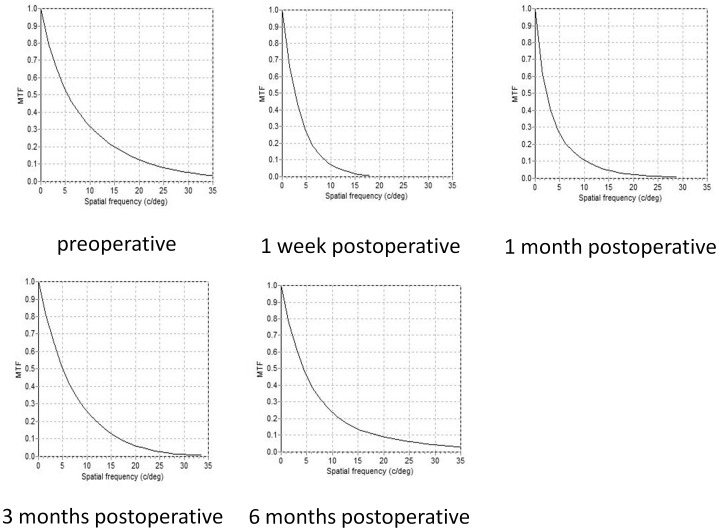
The representative modulation transfer function curves of eyes undergoing femtosecond lenticule extraction preoperatively, and 1 week and 1, 3 and 6 months postoperatively.

Figures 1 and 2 show representative examples of the double-pass images of eyes undergoing FLEx preoperatively, and 1 week and 1, 3 and 6 months postoperatively. Table 2 shows the detailed optical parameters of the eye preoperatively, and 1 week and 1, 3, and 6 months postoperatively. The mean values of the MTF cutoff frequency, the Strehl ratio, the OSI, the OV 100%, 20%, and 9%, and logMAR CDVA, over time were found to be significantly different (p<0.001, ANOVA). In 4 eyes developing interface haze, the OSI was 0.93±0.36, 2.85±1.69, 2.88±2.03, 1.68±1.31, and 1.98±1.43, respectively. At 6 months after surgery, 100% and 89% of eyes had a CDVA of 20/16, and of 20/12.5 or more. We found no significant correlation between the OSI and logMAR CDVA before surgery (Spearman rank correlation coefficient r = 0.068, p = 0.69), and modest, but significant correlations between the OSI and logMAR CDVA 1 week and 1, 3, and 6 months after surgery (r = 0.572, p<0.001 1 week after, r = 0.562, p<0.001 1 month after, r = 0.542, p<0.001 3 months after, r = 0.540, p<0.001 6 months after).

**Table 2:** Time course of the detailed optical parameters of the eye.

	Preoperative	Postoperative (Dunnett test)							
		1 week	P value	1 month	P value	3 months	P value	6 months	P value
MTF cutoff frequency (cpd)	32.92±12.48	19.68±9.17	<0.001	21.22±10.35	<0.001	25.98±10.48	0.02	27.42±9.07	0.08
Strehl ratio	0.19±0.07	0.13±0.05	<0.001	0.13±0.05	<0.001	0.16±0.05	0.03	0.16±0.04	0.02
OSI	0.90±0.52	2.38±1.77	<0.001	2.07±1.34	<0.001	1.44±0.83	0.22	1.26±0.68	0.61
OV 100%	1.10±0.41	0.66±0.31	<0.001	0.71±0.34	<0.001	0.87±0.35	0.02	0.92±0.29	0.11
OV 20%	0.80±0.34	0.49±0.22	<0.001	0.53±0.27	<0.001	0.62±0.25	0.02	0.66±0.23	0.03
OV 9%	0.48±0.21	0.32±0.15	<0.001	0.33±0.18	<0.001	0.39±0.15	0.07	0.39±0.13	0.02
LogMAR CDVA	0.23±0.06	−0.16±0.11	<0.001	−0.19±0.10	0.12	−0.20±0.08	0.22	−0.20±0.08	0.47

cpd = cycles/degree, OSI = objective scattering index, logMAR = logarithm of the minimal angle of resolution, OV = OQAS value, CDVA = corrected distance visual acuity.

## Discussion

In the present study, our results demonstrated that FLEx induced a transient decrease in the MTF cutoff frequency and the Strehl ratio and a transient increase in the OSI in the early postoperative period, but gradually recovered with time. We should be aware that these optical parameters did not completely recover their preoperative levels up to 6 months after surgery, although there were no significant differences in these parameters, except for the Strehl ratio, OV20%, and OV9%, preoperatively or 6 months postoperatively, and all eyes had a CDVA of 20/16 or more 6 months postoperatively. Our results also showed that the OSI was significantly correlated with logMAR CDVA at all postoperative times. It is suggested that this transient degradation in optical quality associated with an increase in intraocular scattering may lead to a slight delay of CDVA recovery in the early postoperative period, but not before surgery. As far as we can ascertain, this is the first study to objectively assess the detailed optical quality of the eye, including the intraocular scattering, after this novel surgical approach. Actually, it has been demonstrated that there was a tendency for a slight delay of visual acuity recovery in the early postoperative period (especially 1 week postoperatively), which is characteristic of this surgical approach. [11], [12], [15]–[17] In this study, the postoperative OSI in eyes developing interface haze was higher than that in the entire population. We assume that mild or clinically insignificant interface haze formation, which was specific for the femtosecond laser processing of the cornea, causes a transient increase in the intraocular scattering after FLEx, although this interface haze resolved with time. Shah et al [11] and Vestergaard et al [16] demonstrated that the initial visual recovery after FLEx was slower than that after femtosecond LASIK. Heichel et al [26] reported that FLEx procedure is capable of creating corneal lenticules of predictable quality, but that the optimization of laser parameters as well as surgical technique is required to improve the regularity of the corneal stromal bed. Kunert et al [27] stated that the surface regularity index decreased as pulse energy increased, and that cases of interface haze were uncommon, since they began to apply lower energies. We previously showed that the efficacy of FLEx was slightly better than that of other previous studies, possibly because of the slightly lower myopic correction and the use of the newer-type femtosecond laser with a 500 kHz repetition rate. [15] We believe that this information was clinically meaningful because further refinement of the laser energy settings, even with the use of the newer-type femtosecond laser, is necessary to improve the visual outcomes, especially in the early postoperative period. We previously examined several optical parameters after posterior chamber phakic intraocular lens (ICL^TM^, STAAR Surgical, Nidau, Switzerland) implantation, and obtained the following values 3 months after ICL implantation: the mean MTF cutoff frequency, 28.69±8.59 cycles/degree; Strehl ratio, 0.17±0.04; OSI, 1.06±0.48; OV100%, 0.96±0.29; OV 20% 0.83±0.31; and OV9%, 0.83±0.32. [28] It is suggested that these optical parameters in ICL-implanted eyes are slightly better than those in post-FLEx eyes in the current study, possibly resulting from a smaller increase in the number of higher-order aberrations and intraocular scattering, and a smaller decrease in retinal magnification after ICL implantation. It would also be of interest to assess the detailed optical quality parameters of the eye after LASIK. We are currently conducting a new study to compare these parameters after FLEx and LASIK.

The OQAS, which is designed on the basis of the asymmetric pattern of the double-pass technique, [18], [19] with different entrance and exit pupil sizes, enabling the detection of both symmetric and asymmetric aberrations, is the only currently available instrument for objectively measuring optical quality in a clinical setting. [20] In addition to optical quality measurements, the system also provides an objective estimation of intraocular scattering. [21], [22] This system allows direct objective measurement of the effect of optimal aberrations and the loss of ocular transparency on the optical quality of the human eye. It has been demonstrated that the device has good repeatability, and that the realignment of the eyes does not impose any additional variation on the measurements. [29], [30] We also confirmed that the mean difference between two measurements with this device (±95% limits of agreement) was 1.02±3.64 cycles/degree (−6.10 to 8.15 cycles/degree) for MTF cutoff frequency, 0.00±0.03 (−0.07 to 0.06) for Strehl ratio, and −0.02±0.17 (−0.35 to 0.32) for OSI. [28] Accordingly, we believe that this device offers reasonable repeatability in the clinical evaluation of the optical quality of the eye.

There are at least two limitations to this study. One is the limited amount of data and the relatively short follow-up. We evaluated the optical quality parameters for up to 6 months postoperatively, when the optical quality of the cornea would have stabilized according to the wound-healing and biomechanical responses of the cornea in a clinical setting. Therefore, at present, we cannot refute the possibility that the optical quality including intraocular scattering was continuously recovered thereafter. A study of longer duration in a larger number of patients is required to clarify this point. Another limitation is that we compared the OSI only with high contrast acuity under photopic conditions. To better understand the exact relationship between intraocular scattering and visual performance after FLEx, it would also be beneficial to assess low contrast acuity as well as visual acuity under mesopic conditions.

In conclusion, our results may support the views that FLEx induced a transient decrease in optical quality associated with an increase in early postoperative intraocular scattering-an increase possibly due to mild interface haze formation-but gradually recovered with time; and that the OSI was significantly correlated with corrected visual acuity at all postoperative times. It is suggested that this transient degradation in optical quality in relation to an increase in intraocular scattering may result in a slight delay of CDVA recovery in the early postoperative period.
